# Electro-optical Study of the Anomalous Rotational Diffusion in Polymer Solutions

**DOI:** 10.1021/acs.macromol.2c01461

**Published:** 2023-01-12

**Authors:** Sergio Martín-Martín, María del Mar Ramos-Tejada, Antonio Rubio-Andrés, Ana B. Bonhome-Espinosa, Ángel V. Delgado, María L. Jiménez

**Affiliations:** † Department of Applied Physics, School of Sciences, University of Granada, 18071Granada, Spain; ‡ Department of Physics, Linares Higher Polytechnic School, University of Jaén, 23700Linares(Jaén), Spain

## Abstract

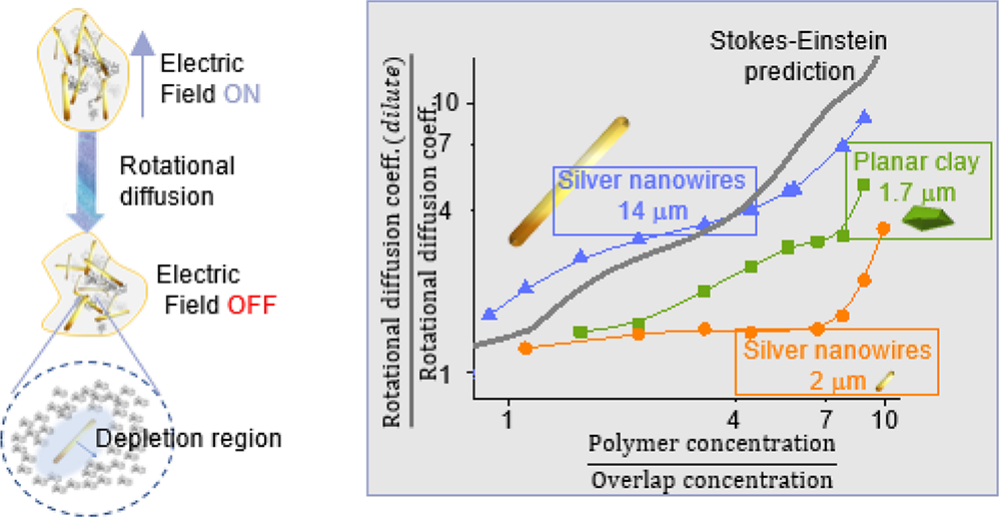

Brownian diffusion of spherical nanoparticles is usually exploited to ascertain the rheological properties of complex media. However, the behavior of the tracer particles is affected by a number of phenomena linked to the interplay between the dynamics of the particles and polymer coils. For this reason, the characteristic lengths of the dispersed entities, depletion phenomena, and the presence of sticking conditions have been observed to affect the translational diffusion of the probes. On the other hand, the retardation effect of the host fluid on the rotational diffusion of nonspherical particles is less understood. We explore the possibility of studying this phenomenon by analyzing the electro-orientation of the particles in different scenarios in which we vary the ratio between the particle and polymer characteristic size, and the geometry of the particles, including both elongated and oblate shapes. We find that the Stokes–Einstein relation only applies if the radius of gyration of the polymer is much shorter than the particle size and when some repulsive interaction between both is present.

## Introduction

The study of the behavior of nanoparticles in polymer solutions has attracted much attention because it may help to understand and manipulate diverse systems with biomedical and nanotechnological applications such as cells, bloodstream, drug delivery systems, or membranes, among others. In order to reach this goal, there is still much work to be done on effects related to the geometry of the particles, the relation between their size, and that of the polymer coils, to mention a few.

Because the Brownian diffusion of probe particles is governed by the viscous properties of the medium, its analysis is used in microrheology studies of complex media, and it has turned out to be an extraordinary tool to explore the nanoscale dynamics in different environments such as biological media.^1−8^ In these studies, the diffusion of particles is monitored, and the Langevin equation is used to obtain from it the rheological properties of the medium.^9^ In this sense, much work has been done to understand the relation between the translational diffusion of micro- and nanoparticles and the viscous response of the solution, coming to the conclusion that Stokes–Einstein (S–E, hereafter) diffusion only occurs if the particle size is large compared to the typical length scales of the heterogeneities of the solution. In such a case, the diffusion times of the particles are typically orders of magnitude larger than the relaxation times of the polymer, and hence, no information about the dynamics of the polymer can be extracted: the medium can be considered as a continuous soup. In contrast, particles that are much smaller than the polymer mesh do not interact with the coils and are expected to diffuse as in polymer-free solutions. Hence, the gold rule in microrheology is that particles must greatly exceed the size of the polymer, characterized by both its radius of gyration *R*_g_ and correlation length ξ. The latter is a measure of the average distance between two monomers of different polymer coils, related to the polymer volume fraction *c* as^10^
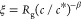
1where β = 0.75 for good solvents and c* is the overlap volume fraction, that is, the ratio between the volume occupied by a single coil (*M*_w_/ρ, where ρ is the density of the polymer and *M*_w_ its molecular weight) and that of a sphere of the size of the radius of gyration:
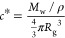
2For dilute solutions (*c* < c*) polymer coils are separated and can be characterized by their radius of gyration. On the other hand, when the volume fraction of the polymer solution is larger than c*, then there is more than one coil per gyration sphere: the coils overlap with each other, and because they are not separated entities, the correlation length turns out to be more relevant than the gyration radius.

In many *in vivo* and *in vitro* systems, where inorganic particles, virus, or bacteria move across a complex biological medium,^1,2,5−7,11−13^ particles are comparable to either the correlation length or the radius of gyration of the host fluid. In this case, a new subdiffusive regime appears, where the mean square of the random walk of particles scales as ⟨*r*^2^⟩ ∝ *t*^α^, with α < 1.^14^ Superdiffusive behavior (1 < α < 2) may also take place, where the dynamics is active or energy consuming, as in particle diffusion in bacterial bath.^15^ For instance, in refs (16−18) superdiffusive behavior was also observed in the rotational diffusion dynamics of active particles. Faster diffusion is also reporter in ref (19), where rotational diffusion of passive particles is observed in a DNA coils solution that is out of equilibrium. All these effects can be more or less successfully explained by different models, such as (i) obstruction models, applied to systems with diffusion times shorter than the polymer relaxation times, and in which the polymer chains are treated as static obstacles that limit the particles movement; (ii) hydrodynamic models, in which the polymer is considered as a collection of drag centers; and (iii) hopping models in which the particles are supposed to be trapped in a cage, diffuse normally inside, and when the fluctuations of the flexible coils open a passage between cages, they can diffuse from one cage to its neighbor.^14,20−22^

On the other hand, not only the geometry but also the affinity of the polymer for the particle surface can influence the particle velocity. Accordingly, sticky and nonsticky pictures have been explored by simulation, scaling theory, and experiments on Brownian motion.^6,20,23−27^

Translation is always accompanied by rotational diffusion, and this has also been demonstrated to be a convenient tool for microrheology determinations. As in the first case, microrheology is based on the S–E relation between the rotational diffusion coefficient and the rheological properties of the continuous media. Hence, works on this subject focus on particles larger than the length scale of the mesh of the complex solution in order for the continuous medium approach to be valid.^28−31^ Compared to microrheology techniques based on translational diffusion, some advantages have been pointed out for the rotational counterpart, for instance, the possibility of exploring systems with large elastic moduli,^9^ where particles may be partially quenched, as it happens in confined geometries,^32,33^ and may be applied in systems with high turbidity.^10^ There are also some approaches providing simultaneous access to translational and rotational diffusion.^34^

In contrast, anomalous rotational diffusion of particles with size similar to that of the heterogeneity in the solution appears much less analyzed. The studies reported in the literature focus on the role of particle geometry. For example, in ref (35) the comparison between translational and rotational microrheology of gels and polymer solutions led to the conclusion that translation and rotation probe the mechanical response of the matrix in different ways, exciting shear and compression modes in the first case and only the shear mode in the second case. Further discrepancies have been found between the S–E rotational diffusion and experimental results,^10,36^ in general associated with the relation between particle and polymer sizes. Finally, while the effect of electrostatic interaction between particle and polymer has been proved to affect the diffusion of particles,^3,4^ as far as we know, the effect on its rotation has not yet been explored.

Rotational diffusion of nonspherical particles is a more complex case because it probes larger deformations of the surrounding solution than the spherical entities. Also, many biological species moving in complex environments have an asymmetric shape. While a stiff virus such as TMV (tobacco mosaic virus) has been shown to diffuse in some polymer solutions according to the S–E relation,^37^ anomalous diffusion has also been demonstrated, leading to the conclusion that not only the size but also the anisotropy is a key factor.^38^

Rotation can be explored by different experimental techniques, depending on the particle properties, including light streak tracking,^9,39^ depolarized light scattering,^35,37^ or magnetic susceptibility. The main drawback of microscope techniques is that they are restricted to large enough microparticles because the rotational diffusion of smaller particles cannot be precisely monitored. On the other hand, with scattering techniques both rotational and translational diffusions are coupled in the response, having the first one a significant impact on the DLS response, and hence this technique still faces experimental challenges.^40−45^

When particles are not spherical, they can be oriented by an electric field, and this can be followed either by microscope observations, or by electro-optic techniques. Again, microscope determinations are restricted with respect to particle size and shape. The electrically aligned particles produce the so-called electric birefringence (EB hereafter) effect, that is, a difference between the refractive index of the system in the directions parallel and perpendicular to the applied electric field. This is proportional to the degree of particle alignment, and, accordingly, rotational diffusion can be monitored by the EB signal. This procedure can be used for a broad range of particle sizes, so it has been a standard technique for the study of nanoparticles and polymers orientation in aqueous solution.^43,46−48^ It is a versatile method, since any micro- or nanoparticle in the fluid can be oriented by the application of alternating electric fields of a suitable frequency, also, we can neglect any effect of settled particles and/or their interaction with walls, and have the advantage of measuring the response of a large amount of particles. In the context of microrheology, this technique has not been yet exploited. Also, small displacements from the equilibrium configuration can be detected by measuring the electric birefringence, this making it possible to examine the medium by only slightly perturbing it.

In this work we use the EB technique to probe anomalous rotational diffusion of particles with different sizes, geometry and surface charges in solutions of polymer. We show that for short polymer coils anomalous diffusion is observed for large particles, which can be attributed to electrostatic interaction. On the other hand, the depletion model can account for the results obtained with long polymer coils, provided that large depletion areas are considered.

## Theoretical Background

When subjected to an external electric field, an electric dipole is induced in nonspherical particles, this promoting the orientation of their longest axis along the field direction. The degree of alignment can be quantified by the orientational order parameter of the system *S*, defined as
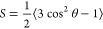
3θ being the angle between the field that produces the orientation and the symmetry axis of the particle. When particles are aligned, the system becomes birefringent, so that the refractive index in the direction parallel to the field (*n*_∥_) differs from that in the perpendicular direction (*n*_⊥_). The birefringence, i.e., the optical anisotropy Δ*n* = *n*_∥_ – *n*_⊥_, is related to the orientational order parameter as^49^
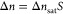
4where Δ*n*_sat_ is the saturation birefringence, that is, the value of Δ*n* attained when particles are perfectly aligned.

In the case of flexible coils such as polymers, they can be deformed by the action of the electric field. However, it has been demonstrated that for moderate field strengths and high frequency the polymer is not perturbed.^50,51^ Furthermore, in ref (19) a synergy was observed between polymer stretching and particles alignment, but this effect was found to disappear at frequencies above 1–10 kHz, where the polymer is not perturbed.

When the applied electric field is switched off, the system takes a time to randomize. For a polydisperse system in a purely viscous and continuous medium, randomization occurs through a rotational diffusion process, which typically produces the decay of the electric birefringence as a stretched exponential function:^52^
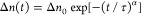
5where Δ*n*_0_ is the electric birefringence when the field is switched off, α is the stretched exponent accounting for the polydispersity of the sample, and τ is the relaxation time, related to the rotational diffusion coefficient Θ through
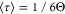
6where , Γ is the Euler gamma function, and Θ depends on the particle geometry and the rheological properties of the medium:
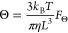
7where *k*_B_ is the Boltzmann constant, *T* the absolute temperature, η the viscosity of the host fluid, *L* the length of the particles, and *F*_Θ_ a geometrical factor that depends on the shape of the particles.

## Experimental Section

### Materials

We have used five types of particles for this study: commercial silver nanowires (Agnws), synthesized silver nanowires (L-Agnws), sodium montmorillonite platelets (NaMt), gibbsite platelets, and elongated silica particles.

Agnws were purchased from PlasmaChem (Germany), and the rest of chemicals were purchased from Sigma-Aldrich (USA). The nanowires were previously characterized by TEM. The mean length of their distribution is 2.0 ± 0.9 μm. They have a polyvinylpyrrolidone ((C_6_H_9_NO)_*n*_, PVP) thin coating that gives them a small negative charge, preventing their aggregation.^19,43^

The preparation of L-Agnws (average length 14 μm) was performed following the procedure in ref (53). NaMt was obtained by homoionization of bentonite (purchased from Sigma-Aldrich, USA). The resulting particles are lamellar with negative charge on their faces and pH-dependent charge on their edges. They were characterized by environmental scanning electron microscopy. Their average diameter is 1.7 ± 0.6 μm.^54^ Both gibbsite platelets (mean diameter (±S.D.) 260 ± 70 nm and thickness of 6.20 ± 0.08 nm) and silica spherocylinders (average length (±S.D.) 430 ± 50 nm and average diameter 190 ± 40 nm) were synthesized according to the procedure described in refs (55 and 56). Further details on the systems preparation can be found in the Supporting Information.

The polymer used was poly(ethylene oxide) (PEO), also known as poly(ethylene glycol), one of the few polymers approved by the FDA for clinical use. PEO compounds are nontoxic and nonimmunogenic as well as soluble in both water and polar solvents.^57^ For this reason, it has been chosen as a model for the development of passive microrheological techniques. Two molecular weights of PEO were investigated: PEO35k with 35 kDa (*R*_g_ = 9 nm and overlap critical concentration c* = 1.58% w/w) purchased from Fluka (Germany) and PEO4M with 4 MDa (*R*_g_ = 152 nm and c* = 0.045% w/w) purchased from Sigma-Aldrich (USA). This is a weakly negatively charged polymer soluble in water.

Calf thymus DNA (CT-DNA, 15 kpb, *R*_g_ = 326 nm and c* = 0.011% w/w) was purchased from Merck, Germany (D1501). In order to disperse the dry fibers of DNA, they were kept in water overnight without stirring. Afterward, volumes of these solutions are mixed with the desired particle suspensions to obtain DNA/particle bidisperse systems. Gentle agitation was sufficient to obtain a homogeneous particle–polymer system. The dispersions were homogeneous, and we checked under 100× microscopy that no aggregation occurs even when the electric field is applied.

The systems under study were immersed in 1 mM NaCl and 0–4% w/w of PEO35k or 0–0.45% w/w of PEO4M.

### Methods

Rheological measurements were performed using a rotational rheometer (Haake MARS III, Thermo Scientific, UK) and thermostated at 15 °C by water circulation. The concentric cylinders geometry (CCB16 DIN/SS) was used. Several strain rate ramps were performed for each polymer solution at different concentrations in order to study the elastic regime and to know the macroscopic viscosity of polymeric solutions. In each test for each sample, the strain rate was increased from 0.1 s^–1^ to 200 s^–1^.

The birefringence measurements were performed with a homemade electro-optical setup consisting of a low-power He–Ne laser beam (Laser Products 05-LHP-151, USA), a polarizer at 45°, the sample to be measured in a quartz Kerr cell (Starna Scientific, UK), a quarter-wave plate, an analyzer, and finally a photodiode connected to an oscilloscope. On the other hand, sinusoidal electric pulses of 1 MHz are applied to the suspension through vertical stainless steel electrodes. A commercial generator (Tektronix AFG 3101, USA) and a low-frequency amplifier (Piezo Systems Inc. EPA-104, USA) are used. All the optical plates and the photodiode were purchased from Edmund Optics, UK. The setup has a water circuit to thermostat all the suspensions at 15 °C. This equipment is described in more detail elsewhere.^54,58,59^ The birefringence is obtained from the change in light intensity collected by the photodiode upon application of the field.

The dynamics of orientation was also visually characterized with an inverted microscope (Olympus Iberia, Spain) equipped with a homemade cell with two aluminum electrodes of 1 mm spacing.

The electrophoretic mobility *u*_e_ of the particles was determined by dynamic light scattering (Malvern Zetasizer NanoZS, Malvern Instruments, UK). From these measurements, the zeta potential ζ was calculated by modeling the particles as infinitely long cylinders (in the case of the wires), prolate spheroids (silica), or oblate spheroids (in the case of NaMt of gibbsite) and making use of the appropriate models for these geometries.^60,61^

## Results and Discussion

### Rheology of PEO

**Figure 1:**
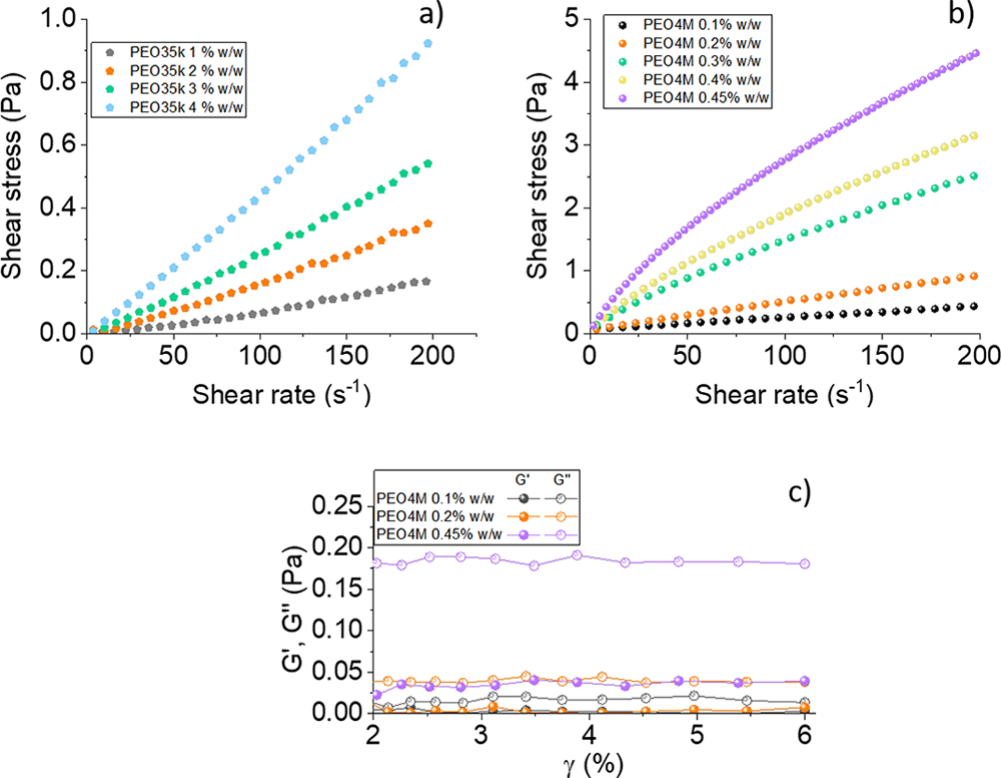
Shear rate–shear stress ramps of PEO35k (a) and PEO4M (b) at the concentrations indicated. (c) Elastic and viscous moduli of PEO4M as a function of shear strain, measured at 1 Hz.

In Figure 1a,b we show the shear rate–shear stress ramp of both PEO35k and PEO4M at different polymer concentrations. We can see that PEO35k has a Newtonian behavior in the whole concentration interval examined in this work. In Figure 1c we observe a finite value of the elastic moduli of PEO4M at the largest PEO4M concentration. These results are in line with those reported in ref (62). In ref (63) it was shown that viscoelasticity has some effect on the rotational diffusion of nanoparticles. It was reported the existence of a yield electric field below which there is no electro-orientation; in addition, such field increases with the elastic modulus of the host solution. On the other hand, the rotational randomization after switching off the electric field was observed to be related to the modulus of the viscosity. Such effects were observed for elastic moduli about 0.2 Pa, which is 1 order of magnitude larger than the largest value in the case of PEO4M. Hence, we can consider that the polymer solutions studied in this work have a Newtonian behavior.

The shear stress ramps in Figure 1a,b also give the order of magnitude of the relaxation time of the polymer. From a purely phenomenological point of view, this time can be calculated from the viscosity data at the characteristic time at the intersection of the constant Newtonian viscosity (measured at low shear rate) with the power law fit in the shear-thinning region (measured at higher shear rate).^64^ In ref (62) it was reported that the relaxation times of PEO range from 0.01 to 0.05 s in the case of PEO4M and from 1 to 30 ms in the case of PEO40k. In the majority of the studied concentrations, we do not observe shear thinning in the shear rate range examined (Figure 1a,b), this meaning that the relaxation time is below 5 ms. On the contrary, we detect shear thinning in the case of PEO4M at the largest concentrations, this occurring at shear rates above 25 s^–1^; hence, in these cases, the relaxation time of the polymer can reach 0.04 s, in line with the results in ref (62).

### Electro-orientation Dynamics of Nonspherical Particles

**Figure 2:**
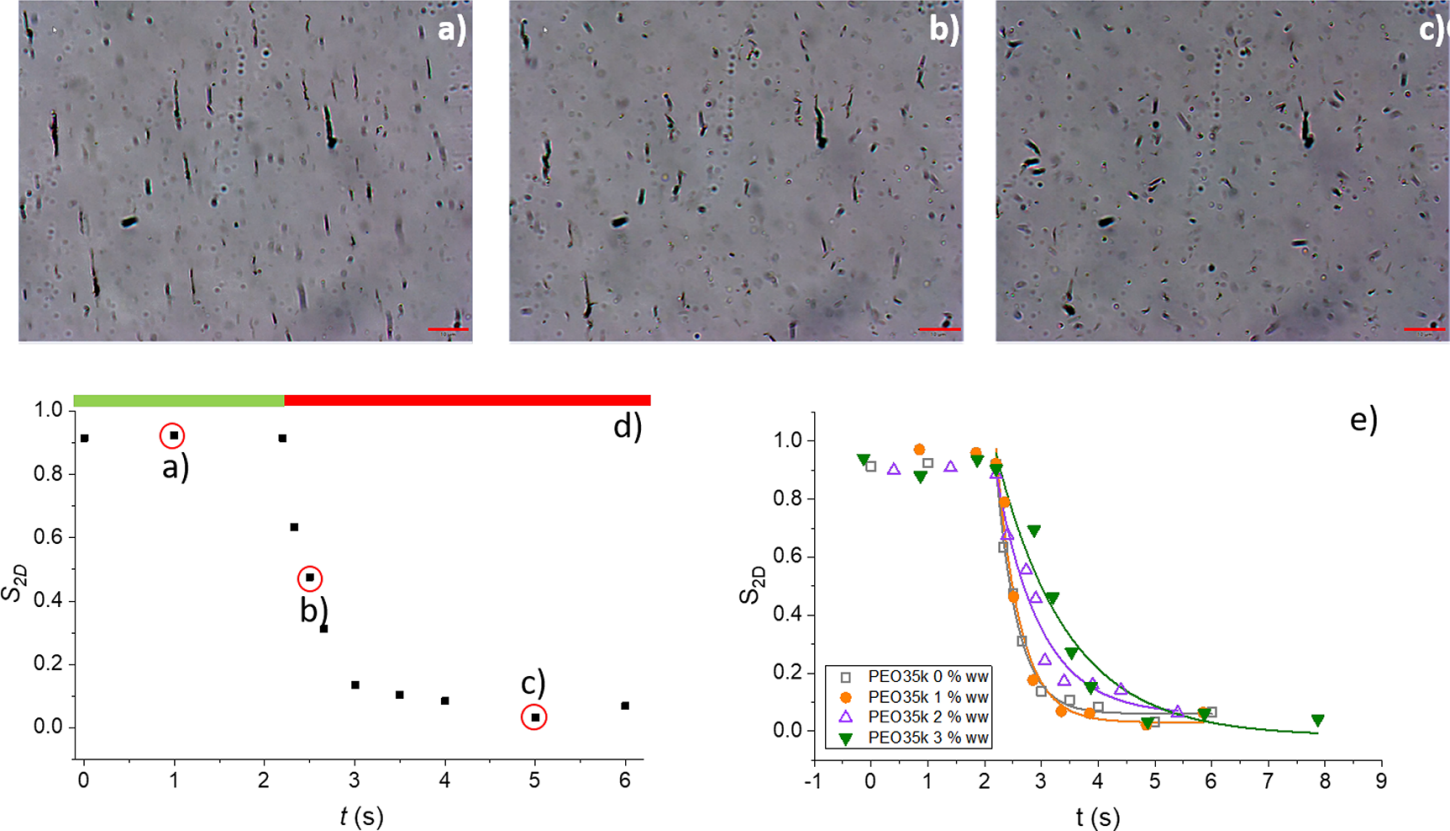
Microscope pictures of Agnws immersed in a water solution at 15 °C and subjected to a sinusoidal electric field (*E* = 10 V/mm and 1 MHz) before (a) and after (b, c) switching off the electric field. The red horizontal bar indicates 10 μm. (d) *S*_2D_ as a function of time for the same system. The three selected points correspond to panels a–c. The horizontal green and red bars correspond to the time interval in which the electric field is on and off, respectively. (e) *S*_2D_ as a function of time for the same particles immersed in PEO35k solutions of the indicated concentration. The lines are the best fit to eq 9.

In Figure 2a–c we show a sample of Agnws immersed in water initially subjected to a sinusoidal electric field and at two times after switching it off. We can observe that the particles are initially partially aligned, and after a certain time they become randomly oriented. Note that we can only measure the projection of the particles on the *XY* plane, where just the angle θ_2D_ between this projection and the applied field can be measured. Therefore, instead of calculating the orientational order parameter eq 3, we calculate
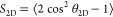
8The differences between this parameter and *S* have been demonstrated to be negligible.^65^ Thus, the electric birefringence dynamics and that of *S*_2D_ must coincide, that is
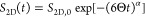
9We measured *S*_2D_ at different stages after switching off the electric field with a partially automatic procedure, in which the contrast between particle and medium was manually enhanced and a software was used to measure the particle orientation. Figure 2d is an example. Note that despite the large sampling time interval, the results can be fitted to a stretched exponential from which the rotational diffusion coefficient of the particles can be found. This is shown in Figure 2e, where the electro-orientation decay after switching off the electric field is observed for different PEO35k concentrations.

### Electric Birefringence Results. Effect of Particle Size and Geometry

#### Characteristic Time of the Rotational Diffusion

**Figure 3:**
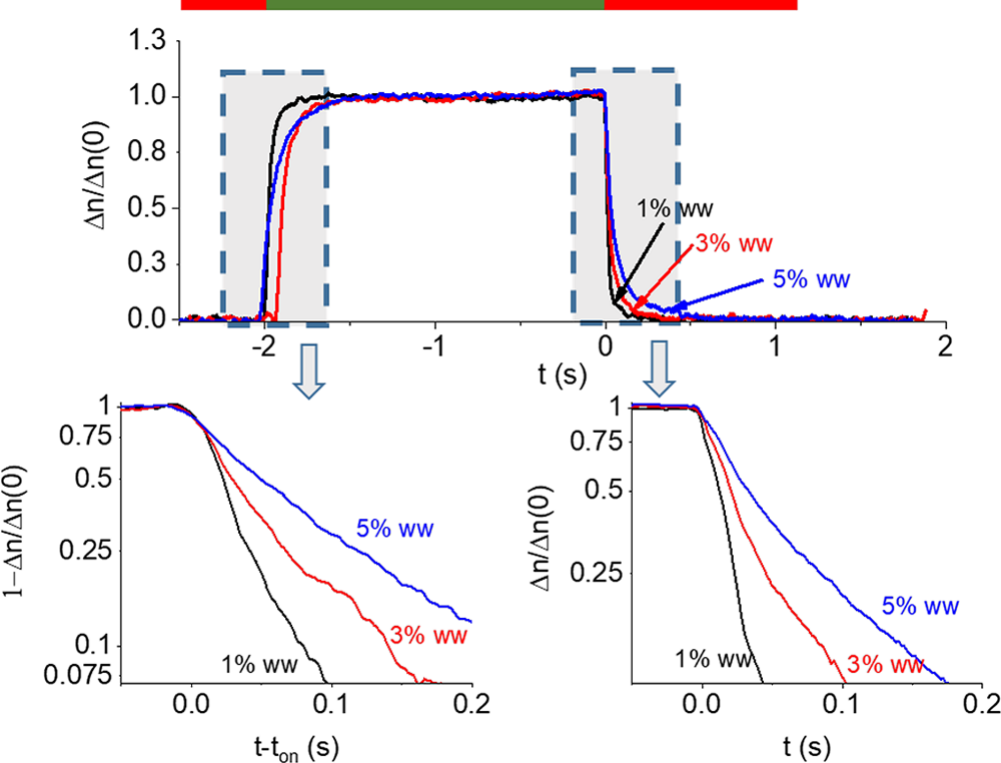
(top) Electric birefringence of a mixture of gibbsite nanoplates immersed in the indicated concentrations of PEO35k. Red and green bars indicate electric field off and on, respectively. (bottom) Details of the rising (left) and decay (right) parts of the electric birefringence.

Figure 3 shows the typical response curve of gibbsite platelets with PEO35k. The intensity of the electric field is in all cases below 10 V/mm, for which the system obeys the Kerr law.^48,54,58^ In this regime, the Peclet number is *Pe* ≪ 1, this ensuring that the forced particle will not introduce a significant distortion on the host fluid.^66^ We can observe that the decay is slower when the polymer is present, as also observed for the rest of particles and polymers.

**Figure 4:**
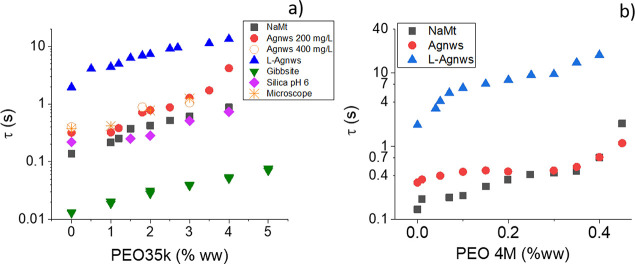
Characteristic time of the electric birefringence decay of systems made of the indicated particles immersed in the PEO35k (a) and PEO4M (b) solutions. The microscope observations were performed with Agnws.

Next, we fit the birefringence decay to eq 5 in order to obtain the characteristic time of the rotational randomization. These times are plotted in Figure 4 for different concentrations of PEO35k and PEO4M. In this plot we add the characteristic time obtained in the microscope experiments, observing a reasonable agreement, this being a confirmation that we are observing the same particle randomization dynamics. However, note that the sampling time interval is much smaller in birefringence experiments, and hence, more accurate results can be obtained.

As expected, the characteristic time τ in Figure 4 increases as the polymer concentration rises, a result of the polymer-induced retardation. τ values larger than 0.1 s are obtained, which are more than 1 order of magnitude longer than both the entanglement/disentanglement times^62^ and the relaxation times of the polymer estimated above. This indicates that the inertia of the polymer relaxation does not play a significant role on particle rotation.

Note also that the characteristic time does not depend on the concentration of Agnws. Furthermore, we checked that the electric birefringence is proportional to the particle concentration in this concentration range, this ruling out any possible contribution of particle–particle interaction. On the other hand, the characteristic times of the particles in a water solution (eqs 6 and 7) and the appropriate geometry factors *F*_Θ_ in every case^43^ can be used to determine the characteristic size of the particles. In the Supporting Information we can see that particles have different degrees of polydispersity. The impact of polydispersity on the rotational diffusion has been deeply investigated in the past. In refs (52 and 67) it was demonstrated that the stretched exponential function accurately simulates the orientational randomization process of polydisperse systems. In fact, the characterization of the size with electric birefringence experiments of the particles studied in the present work has already been successfully used in refs (43 and 58) where it was shown that the particle size is well predicted by the rotational diffusion decay fitted to a stretched exponential.

A good agreement between the particle sizes obtained from both TEM microscopy and EB experiments is also observed in our case (see Table 1). In the case of L-Agnws the sedimentation of the largest particles may be the reason for the large difference between both results.

**Table 1:** Characteristic Size of the Particles as Obtained from Microscope Determinations (TEM) and from the Electric Birefringence Decay (EB) as Well as the Stretched Exponential Coefficient α

particle	TEM size (S.D.) (nm)	EB size (nm)	α
gibbsite	260 (70)	276 ± 5	0.7–0.8
NaMt	1700 (600)	1500 ± 20	0.6–0.7
silica	430 (50)	470 ± 3	0.5–0.6
Agnws	2000 (900)	1680 ± 13	0.6–0.7
L-Agnws	14000 (1400)	5100 ± 200	0.8–0.9

#### Estimation of the Rotational Diffusion Coefficient

**Figure 5:**
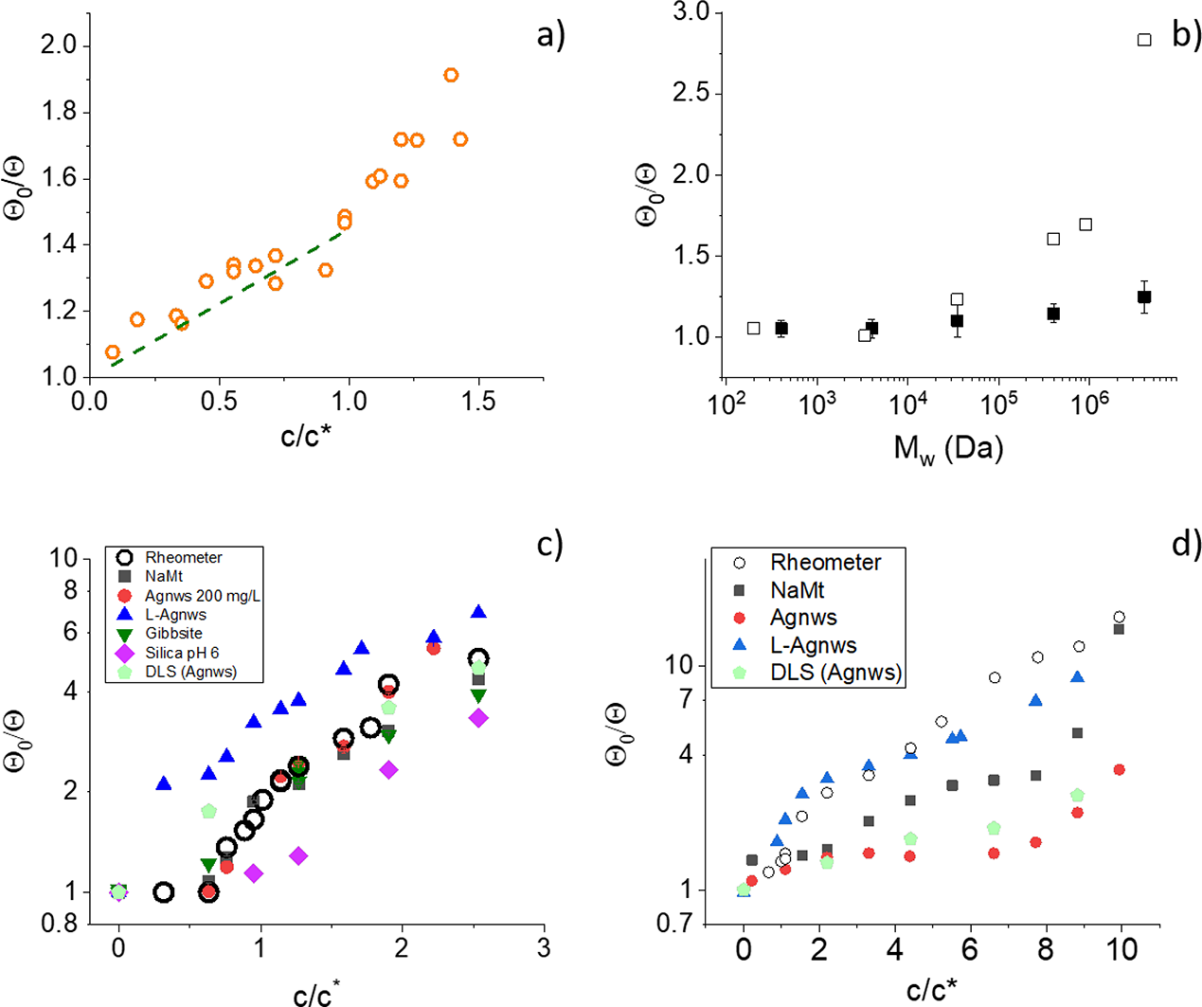
Θ_0_/Θ for Agnws in CT-DNA solution (a), for Agnws in 0.02% w/w PEO solutions of different lengths (b), and for NaMt, Agnws, and L-Agnws immersed in PEO35k (c) and PEO4M (d) solutions. Particle concentration: 200 mg/L. The dashed line in (a) is the macroscopic viscosity (taken from ref (72)). The data labeled “Rheometer” refer to the predictions of Θ_0_/Θ based on the viscosity as measured by the rheometer. The data labeled “DLS (Agnws)” correspond to the predictions of Θ_0_/Θ based on dynamic light scattering measurements of Agnws.

The characteristic times can be used to obtain the rotational diffusion coefficient via eq 6. This coefficient decreases with respect to the value in pure water due to the retardation induced by the polymer. It also depends on the size and geometry of the particles (eq 7). Note that the size may increase by the adsorption of the PEO coils, and this effect has been reported and used for controlling the stability of colloids.^68−71^ Such a soft layer can grow up to tens of nanometers, even for particles with negative surface charge. Hence, its effect on the rotational diffusion coefficient cannot be ruled out. However, for PEO concentrations larger than 0.002% w/w,^71^ the thickness does not increase with polymer concentration, this indicating that the layer is saturated, and hence, the ratio Θ_0_/Θ (where Θ and Θ_0_ are the rotational diffusion coefficients in the polymer solution and in the most diluted one, respectively) is expected to be independent of the size of the particles. This quantity is represented in Figure 5. In all cases we observe an increasing behavior with both the concentration and the molecular weight of the polymer, as expected considering that the viscosity increases with the polymer concentration and molecular weight.

In all cases we have added the expected value of Θ_0_/Θ assuming that the S–E relation is valid and using the rheometer viscosity η normalized by the viscosity of pure water, η_0_. In fact, according to eq 7, in a normal rotational diffusion process, Θ_0_/Θ and η/η_0_ should be identical, and this is experimentally observed in the case of the biopolymer CT-DNA (Figure 5a).

Below the overlap concentration, the radius of gyration is the characteristic size of the polymer, while the comparison between ξ and the particle size must be considered for larger concentrations.^73,74^ In the case of DNA (Figure 5a), *R*_g_ ≈ 300 nm, which is smaller than the particle size. Beyond the overlap concentration, the correlation length is smaller than the radius of gyration of the polymer, and hence, it always fulfills the conditions for S–E application. Note also that in this case the polymer has a large negative charge, so polymer/particle attraction is negligible. In fact, normal diffusion of particles in DNA solutions have been observed by other authors.^75,76^

The effect of the radius of gyration of the polymer is observed in Figure 5b. Note that c* > 0.04% w/w, so we are below the overlap concentration in all the cases shown in this figure. As the polymer length (and hence, *R*_g_) increases, so does the deviation from the values expected from rheometer determinations. Furthermore, in ref (77) it is shown that the breakdown of the S–E relation occurs when the particle size is smaller than the total contour length of the polymer. In our case, this corresponds to polymer of *M*_w_ ≈ 120000–150000, in accordance with results in Figure 5b.

In Figures 5c and 5d we show the effect of the polymer concentration on the rotational diffusion coefficient for both short and long polymers. Normal diffusion is observed for Agnws and NaMt immersed in PEO35k solutions (Figure 5c), while a disagreement between rheometer predictions and EB results is found in the other cases. We also show that DLS measurements (in which *D*_0_/*D* can be obtained, and this can be taken as a measure of Θ_0_/Θ) are in good agreement with EB results. In contrast, in PEO4M solutions, normal diffusion is only observed in the case of the largest particles: L-Agnws.

**Figure 6:**
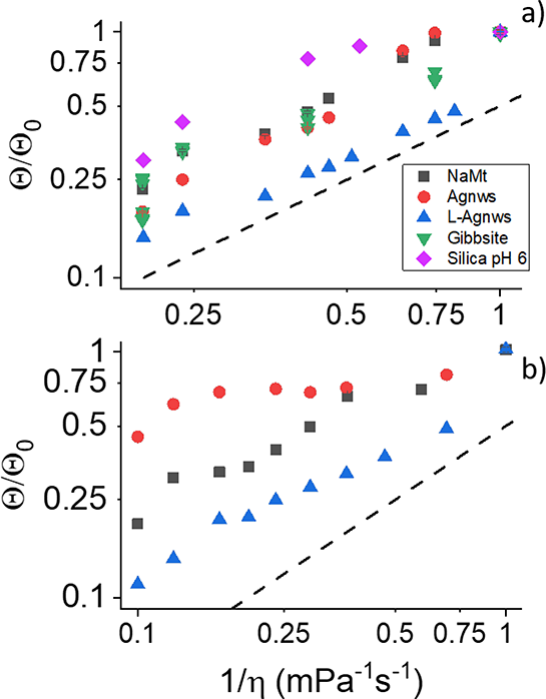
Θ/Θ_0_ for the particles indicated immersed in PEO35k (a) and PEO4M (b) as a function of the inverse of the viscosity of the solution. The lines are parallel to the S–E proportionality relation.

The analysis of the agreement with the S–E relation is better observed in Figure 6, where Θ/Θ_0_ is plotted against the inverse of the viscosity determined with the rheometer. The differences between Agnws and NaMt, of similar size but different shape, are enhanced in this graph, where we can see that the S–E linearity Θ ∝ 1/η is completely lost in the case of Agnws in PEO4M. The same occurs in the case of PEO35k, where oblate gibbsite particles have a normal behavior, but elongated silica particles do not.

#### Effect of the Polymer Correlation Length

**Figure 7:**
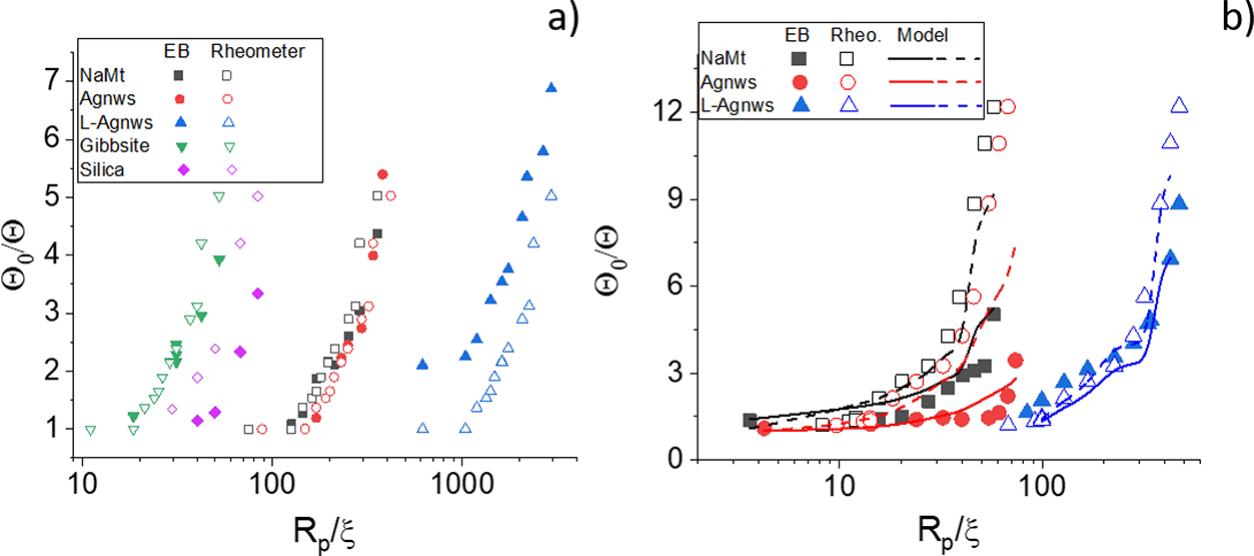
Θ_0_/Θ as a function of *R*_p_/ξ, *R*_p_ being the largest dimension of the particles tested, immersed in PEO35k (a) and PEO4M (b) solutions as a function of *R*_p_/ξ. Solid symbols: electric birefringence determinations. Open symbols: predictions from the experimental macroscopic viscosity. In (b): dashed lines: predictions from the scaling law in ref (74); solid lines: scaling law in ref (74) for a 5 times larger depletion region.

The effect of the ratio between the correlation length of the polymer and the particle size can be appreciated in Figure 7, where the data are represented as a function of *R*_p_/ξ (the *R*_p_ being the largest dimension of the particle). In all cases *R*_p_/ξ > 1, this meaning that particles and polymer should have multiple contacts if the polymer is homogeneously distributed. However, we observe no or small retardation on the diffusion of particles with *R*_p_/ξ < 100. Fast diffusion of spherical probes in polymer solutions has been previously attributed to depletion effects.^36,74,78−80^ As a consequence of the nonhomogeneous polymer distribution close to the particle, the retardation is reduced with respect to the expected value in a continuous phase, and a scaling law is derived for this retardation. Assuming that the fluid surrounding the particle is structured in two layers, one with the viscosity of the solvent and the other with the viscosity of the polymer solution, in ref (74) it was derived the following expression for the retardation factor Θ_0_/Θ:
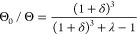
10where δ is the thickness of the depletion layer and λ = η_0_/η (η being the macroscopic viscosity). The predictions of this model for our system are included in Figure 7b (dashed lines). As observed, this effect is not enough to explain our results. The model was developed for rotating spherical particles, while in our case particles are highly nonspherical. Furthermore, in electric birefringence measurements, the system is only slightly distorted from the equilibrium random orientation. This means that particles only rotate a small angle during the measurements, and the medium is little modified. This may be modeled by a thicker depletion layer. According to our data, a 5 times larger depletion region may account for our results (solid lines).

The enhancement of the depletion region may originate from the electrostatic repulsion between the particle and polymer. Even though the polymer has a small negative charge, this may be enough to produce an electrostatic repulsion that deforms the polymer coils around the particles. Such effect was already predicted in ref (81) where electrostatically repulsive polymer–particles mixture was observed both experimentally and with integral equation theory. Further evidence is found in ref (82) for a binary mixture of repulsive particles.

Alternatively, attractive interactions between particle and polymer may have some delay effect.^3,4,83^ This explanation fits with the fact that L-Agnws are slightly positive (see Table 2). On the contrary, it does not occur in the case of the negatively charged Agnws and NaMt immersed in PEO35k or PEO4M and in CT-DNA (see Figure 5a). The attraction between particles and polymer can be interpreted as an increase of the effective hydrodynamic radius.^66^ In fact, a larger thickness of the adsorbed polymer is reported in ref (71) when particles and polymer have opposite charges. This implies that at higher polymer concentration we have larger particles and thus smaller diffusion coefficients than expected for bare particles.

**Table 2:** Electrophoretic Mobility, Zeta Potential, and Electrokinetic Charge of the Particles in 1 mM NaCl Solution^a^

particle	*u*_e_ (10^–8^ m^2^/(V s))	ζ (mV)	surface charge density (mC/m^2^)
gibbsite	–2.5 ± 0.3	–35 ± 4	–2.8 ± 0.3
NaMt	–4.3 ± 0.3	–70 ± 5	–7.2 ± 0.8
silica	–1.3 ± 0.1	–18 ± 2	–1.4 ± 0.2
Agnws	–0.8 ± 0.2	–20 ± 5	–1.5 ± 0.4
L-Agnws	+0.4 ± 0.2	5 ± 3	0.36 ± 0.15

a The models in refs (60 and 61) were used to calculate the ζ potential.

## Conclusions

In this work we show that electro-orientation of nonspherical particles can be used to describe the rotational diffusion coefficient in polymer solutions. Despite this being a phenomenon similar to translational diffusion, we find an unexpected result: when the particle size is less than roughly 100 times the correlation length, the macroscopic viscosity underestimates the rotational diffusion coefficient of the particles. The deviations are larger in the case of elongated particles: the rotational diffusion dynamics in this case seems to be less affected by the presence of the polymer. Comparing NaMt and AgNWs in PEO4M, we see larger deviations in the second case, which fail to obey the proportionality of the S–E relation. The same is observed in the case of elongated silica in PEO35k. On the other hand, both gibbsite and NaMt, although deviating from the predictions of the rheometer, still follow the S–E behavior.

We have shown that the retardation effect of the polymer coils on the diffusion of particles can be well described by depletion models with an enhanced size of the depletion region due to the electrostatic repulsion between the components of the mixture. On the other hand, PEO35k produces an unexpectedly large retardation effect on the long silver nanowires, which can be attributed to the attractive interaction between particles and polymer.

The behavior of tracer particles immersed in complex media is affected by a number of phenomena due to the interplay between particles and polymer coils dynamics. Length scales of particles and polymer coils, depletion, and the presence of sticking conditions have been previously observed to affect the translational diffusion of the probes, and as we demonstrate, they also govern the rotational diffusion. The used experimental method, based on electric birefringence, opens the possibility of exploring more complex systems, relevant in nanotechnology, such as viscoelastic fluids, active particles, or confined media.

## Supporting Information Available

Additional experimental details with the materials, including TEM pictures and size distributions (PDF)
