# Supplementary material for: Electro-optical Study of the Anomalous Rotational Diffusion in Polymer Solutions
Source: Macromolecules. 2023 Jan 12;56(2):518–27. doi: 10.1021/acs.macromol.2c01461 (PMC9879198; doi:10.1021/acs.macromol.2c01461)
Supplement: Supplementary file 1 — ma2c01461_si_001.pdf [file ma2c01461_si_001.pdf]

# Electro-optical study of the anomalous rotational diffusion in polymer solutions

Sergio Martín-Martín,[†] María del Mar Ramos-Tejada,[‡] Antonio Rubio-Andrés,[†] Ana B. Bonhome-Espinosa,[†] Ángel V. Delgado,[†] and María L. Jiménez[*,†]

†*Department of Applied Physics, School of Sciences, University of Granada, 18071, Granada, Spain.*

‡*Department of Physics, Linares Higher Polytechnic School, University of Jaén, 23700 Linares, (Jaén), Spain*

E-mail: jimenezo@ugr.es

**SUPPLEMENTARY INFORMATION FILE**

## Materials

L-Agnws were synthesized following the procedure described in Ref.[1] This procedure provides a sample of polydisperse Agnws ranging between 1 and 15 $\mu$m in length (see Fig. 1a). In order to reduce the polydispersity, the sample was passed through cellulose filters of 3 $\mu$m and 8 $\mu$m pore size. In Fig. 1b we show the histograms of the length (left) and diameter(right).

The synthesis of gibbsite particles is described in.[2] In Fig. 2 it can be seen a TEM picture. The mean diameter ($\pm$ S.D.) $2b = 207 \pm 3$ nm and thicnkess of $2a = 6.20 \pm 0.08 nm$ measured over more than 200 particles.

We follow the protocol in.[3] 0.31 g of CTAB and 0.05 g of Poly(ethylene glicol) (molecular weight 200, Sigma Aldrich) was disolved in 50 mL of Milli-Q water (Millipore, France) at 40

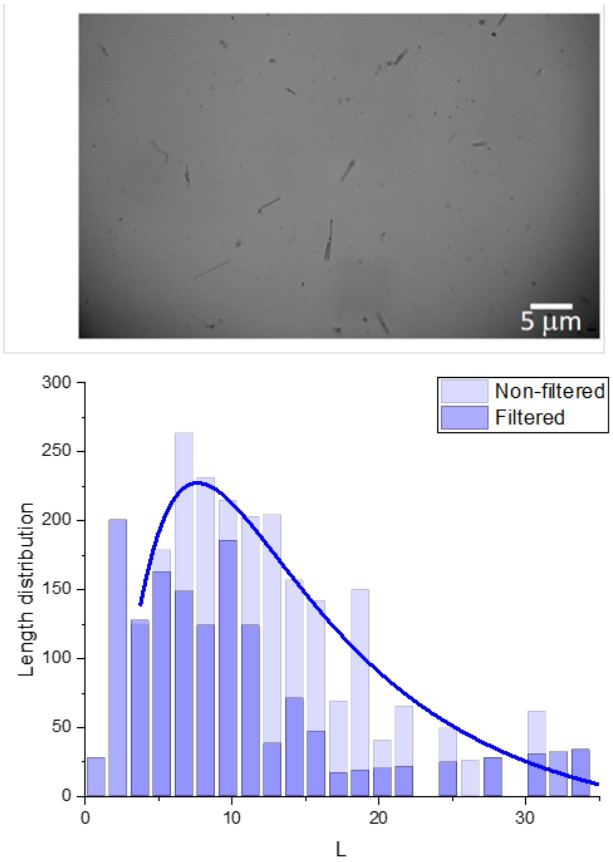


Figure 1: Microscope image and volume distribution of the length of the L-Agnws sample. The line is the best fit to a lognormal distribution.

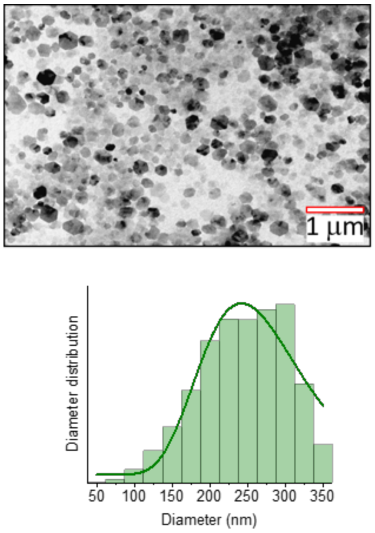


Figure 2: TEM picture of gibbsite particles and volume distribution of its diameter. The line is the best fit to a lognormal distribution.

°C. Then, 2.6 mL of 30% NH4OH (Scharlau) was added. The final step was 1,5 mL of TEOS (tetraethyl ortosilicate, 98%, Sigma Aldrich). We keep the mixture at 40 ° C under magnetic agitation during two hours. We followed a protocol of 4 cycles of centrifugation/redispersion in etanol and water. Once the nanoparticles are dried, we put then at a 50 ° C/min ramp until 600 ° C in a cylindrical oven and aged the powder at this temperature during 1 hour. We obtained a spherocylinders as those shown in Fig. 3. The average length (± S.D.) is 430±50 nm and the average diameter is 190±40 nm.

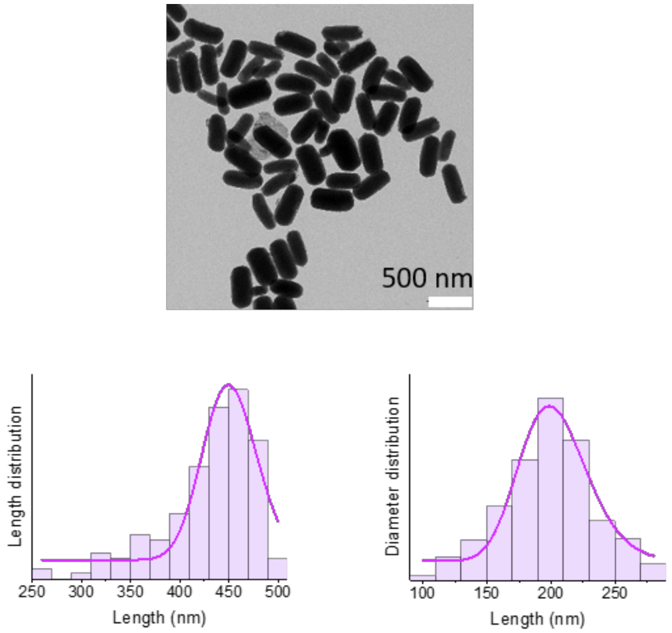


Figure 3: TEM picture of silica particles and volume distribution of its length and diameter. The line is the best fit to a lognormal distribution.

# References


(1) Wang, S.; Tian, Y.; Ding, S.; Huang, Y. Rapid synthesis of long silver nanowires by controlling concentration of $Cu^{2+}$ ions. Materials Letters **2016**, 172, 175–178.

(2) Wijnhoven, J. E. Seeded growth of monodisperse gibbsite platelets to adjustable sizes. Journal of colloid and interface science **2005**, 292, 403–409.

(3) Lin, C.; Song, Y.; Gao, F.; Zhou, X.; Sheng, Y.; Shi, Z.; Zou, H. Luminescent properties and energy transfer of Gd3+/Eu3+ co-doped high uniform meso-silica nanorods. Journal of Luminescence **2015**, 158, 456–463.
